# Near-Surface Seismic Measurements in Gravel Pit, over Highway Tunnel and Underground Tubes with Ground Truth Information as an Open Data Set

**DOI:** 10.3390/s22176687

**Published:** 2022-09-04

**Authors:** Ban-Sok Shin, Luis Wientgens, Marius Schaab, Dmitriy Shutin

**Affiliations:** German Aerospace Center, Institute of Communications and Navigation, Münchener Str. 20, 82234 Wessling, Germany

**Keywords:** seismic exploration, open seismic data set, seismic refraction survey, travel time tomography

## Abstract

In this article, we describe in detail three seismic measurement campaigns based on refraction methods that we conducted at different sites in Bavaria, Germany. The measured data is published as an open data set. The particularity of this data set lies in its available ground truth information about each measurement site. Acquiring seismic data from sites with ground truth information is important for validation of seismic inversion algorithms. Since near-surface seismic field data with ground truth information is rather limited, we anticipate this data set to be a valuable contribution to the research community. For the measurements, three sites have been selected: (1) a gravel pit with a ground water layer, (2) a site above a highway tunnel and (3) a surface over underground tubes. The measurements have been conducted using line arrays of geophones, the Geode Seismograph from Geometrics Inc. and hammer strikes as seismic source. To obtain inversion results a travel time tomography based on first-arrivals within the software SeisImager is used. The inversion results show that we are able to image the ground water layer in the gravel pit, the highway tunnel and partly features of underground tubes. Furthermore, the results coincide with available ground truth information about the measurement sites. This paper summarizes the measurement campaigns and the respective data sets obtained through these campaigns. The data have been published by the authors as an open data set under the license CC BY 4.0 on figshare to make it available to the research community for validation of seismic data processing and inversion techniques.

## 1. Introduction

Acquiring real seismic data from sites with available ground truth information about the subsurface is a challenging task. At measurement sites, there is often insufficient information about the inner structure and materials available. Subsurface features such as material layers or underground anomalies and their respective location within the subsurface are usually not known a priori. However, without information about the ground truth, recorded measurement data only offer limited use when verifying algorithms in their capability to process real data from the field. To obtain more information about the inner structure, techniques such as borehole logging can be used, revealing materials within the subsurface over depth. Another approach is to construct an individual subsurface by, e.g., burying certain anomalies in the ground. This requires substantial effort and planning and is therefore seldom carried out in practice. A different approach is to generate seismic data on a smaller scale in the laboratory [1,2]. In this case, one uses ultrasonic waves and sensors instead of seismic waves and geophones. The employed higher frequency makes it possible to miniaturize the complete measurement setup and to individually design a subsurface structure with certain material properties. However, due to the difficulty of coupling the ultrasonic waves into the material, the choice of subsurface materials is limited. Furthermore, the design and construction of such a miniaturized setup is a challenging task in itself.

Despite the aforementioned challenges, there exist some studies that include seismic surveys with ground truth information. Recent examples include [3,4,5]. In [3] the authors investigate the capability of travel time tomography in combination with full waveform inversion for the inversion of near-surface land seismic data. Here, ground truth information about the P-wave velocities in the subsurface was obtained with the help of multiple 20 m deep boreholes. The authors of [4] performed microseismic data analysis with near-surface seismic data at the Corona Volcano, Canary Islands, Spain area. Here, a near-surface lava tube was mapped using a laser scanner such that ground truth information was available and could be used to verify the analysis results. In [5] the authors conducted near-surface seismic measurements in Lagos, Nigeria, to characterize the site for stable constructions. Ground truth information was obtained using cone penetration and drilling techniques. Results from those surveys are compared to those of the seismic refraction survey. However, in general, the number of near-surface seismic data with ground truth information is rather limited. This becomes particularly apparent when compared to studies considering ground penetrating radar (GPR), another widely used technique for near-surface geophysical imaging; see, e.g., [6,7,8,9,10]. This is due to the fact that the achievable penetration depth of GPR is limited to the cm to m range depending on the frequency of the electromagnetic wave and the respective subsurface material. Hence, GPR surveys usually aim at shallower depths; therefore, taking ground truth samples from the subsurface or constructing ground truth models is less challenging compared to the seismic case. It is therefore of high importance to provide seismic field data that include ground truth knowledge.

Near-surface seismic experiments are commonly performed using techniques from refraction or reflection seismology [11]. Here, a seismic source is used to inject mechanical waves into the ground. An array of geophones is placed nearby that captures reflected and refracted waves from the subsurface. Common seismic sources include hammer strikes, falling weights, explosive sources or seismic vibrators for larger depths. Based on the acquired waveform data, an inversion is performed that aims to estimate the spatial distribution of certain parameters of the subsurface, e.g., velocity, material density. Inversion methods range from simple techniques such as normal move-out [11] to more advanced imaging algorithms such as reverse time migration [12], travel time tomography [13] or full waveform inversion [14,15]. While reverse time migration produces images with respect to the reflectivity properties of the subsurface, travel time tomography and full waveform inversion reconstruct images regarding physical parameters such as velocity and density. In its simplest form, travel time tomography relies on first arrivals of incoming waves. Based on these, travel times are measured and compared to synthetically generated travel times based on a model of the subsurface. Travel times can be synthesized by solving the Eikonal equation. The obtained residual is then used to adapt the subsurface model until a minimum is reached, with the goal of bringing the subsurface model closer to the true model. In contrast with travel time tomography, full waveform inversion exploits the whole seismogram measured at the geophones. More specifically, it compares measured data to synthesized seismograms that are generated by solving the acoustic or elastic wave equation. This again provides residual data that are minimized to obtain a subsurface model close to the true one. Full waveform inversion provides an image with a higher spatial resolution compared to travel time tomography. However, the cost function in full waveform inversion is non-convex and the probability of ending up in a local minimum is high. Therefore, usually, a travel time tomography is performed first to obtain a rough image of the subsurface. After that, full waveform inversion is applied in order to obtain high-frequency details in the image.

### Main Contribution

In this paper, we give a detailed description of the seismic measurement campaigns we conducted to obtain data with ground truth information about the respective subsurface under investigation. We selected three outdoor sites with available ground truth information about the inner subsurface structure. The first place was inside a gravel pit in Planegg, Bavaria, Germany. Here, we had ground truth information about the depth of the ground water layer inside the pit provided by the management site. The next measurement site was above a highway tunnel in Aubing, Bavaria, Germany. The tunnel provides a large reflective body that should be visible in the measurement data. Regarding ground truth information, the approximate tunnel depth from the surface as well as the location where the tunnel started were available. This information was provided by the highway management site. As the final measurement site, we chose a place at the German Aerospace Center (DLR) premises in Oberpfaffenhofen, Germany. Here, we had ground truth information available about the depth of near-surface underground pipes from underground maps provided by the site management. The obtained measurement data have been published as an open data set and can be used to validate seismic data processing and inversion algorithms. In addition, we present the first inversion results for these measurement data with respect to P-wave velocity models and compare these to the available ground truth information.

The paper is structured as follows: In Section 2, Section 3 and Section 4, we describe the measurement setups and respective campaigns in the gravel pit, over the highway tunnel and over the underground tubes. For each measurement campaign, we show and discuss travel time curves and tomographic inversion results based on the measurement data. Section 5 gives details about the structure of the open data set that is published. Lastly, in Section 6 we give a summary and conclusion regarding the described measurement campaigns and respective imaging results.

## 2. Seismic Measurements in Gravel Pit

**Figure 1:**
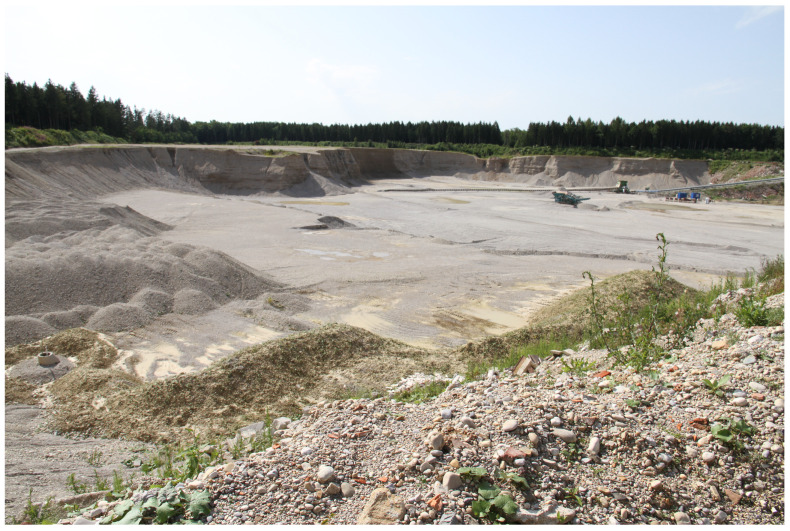
View of the complete gravel pit of Kiesglück in Planegg, Germany.

**Figure 2:**
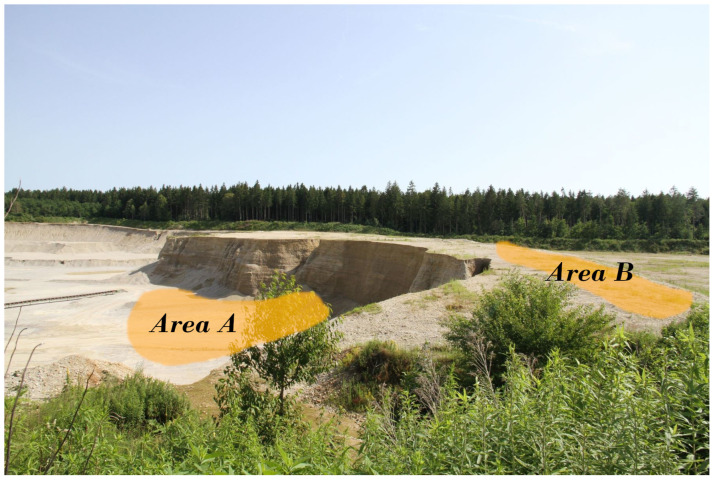
Areas A and B in the gravel pit where measurements have been recorded. Three measurements have been taken inside the pit (Area A) and one on the edge of the pit (Area B).

In this section, we describe in detail the measurement setup in the gravel pit and the obtained inversion results. These measurements were conducted in July 2021 for half a day in the gravel pit of the company “Glück Kieswerk Gräfelfing” in Planegg, Germany [16]. Figure 1 shows a view of the complete gravel pit, which lies several meters below the surface. Figure 2 depicts a site view over the gravel pit, with marked areas where measurements have been recorded. Area A lies inside the gravel pit, while Area B lies on top of the hillside next to the pit.

### 2.1. Measurement Setup

**Figure 3:**
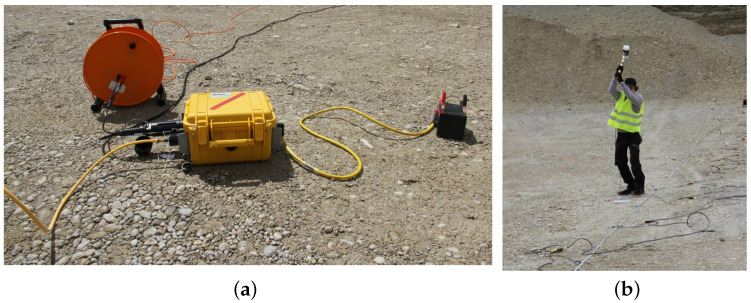
(**a**) Geometrics Geode connected to geophones and a 12 V battery. (**b**) Hammer strike as seismic source.

For the near-surface refraction survey, we used 16 geophones (GS-20DX) from Geospace Technologies with a resonance frequency of 40 Hz spread out in a line array constellation over the surface. We chose a resonance frequency of 40 Hz in order to record reflected and refracted waves with higher fidelity than surface waves which have high amplitudes at frequencies from 5 Hz–30 Hz. Thus, by using 40 Hz geophones, surface waves will distort measurements of the reflected waves less, since their amplitude is lower at this frequency. To process the recorded data, we employ a Geode exploration seismograph from Geometrics, s. Figure 3a. As seismic source, we use a sledgehammer with 4 kg weight. The sledgehammer is struck on an aluminum plate by a person to generate seismic waves in the subsurface, s. Figure 3b. The Geode is connected to a notebook via an ethernet cable. The notebook uses the Geometrics Seismodule Controller software to access the data of the Geode. Furthermore, each geophone is connected to the same cable via clips, and the cable is connected to the Geode. Regarding the hammer signal, a trigger cable is mounted to the hammer via tape and connected to the Geode. When the hammer is struck on the aluminum plate, a trigger signal is produced that initiates the Geode to record the data from the geophones.

### 2.2. Measurements in Area A of Gravel Pit

**Figure 4:**
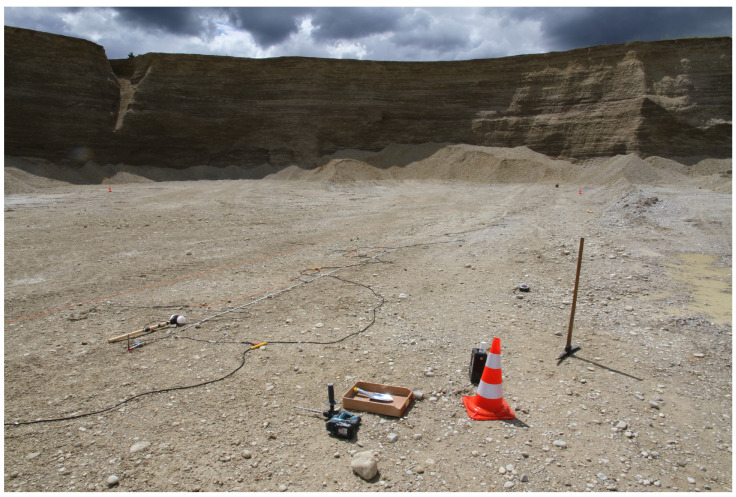
Closer view of Area A inside the gravel pit. We measured perpendicular to the hillside.

**Figure 5:**
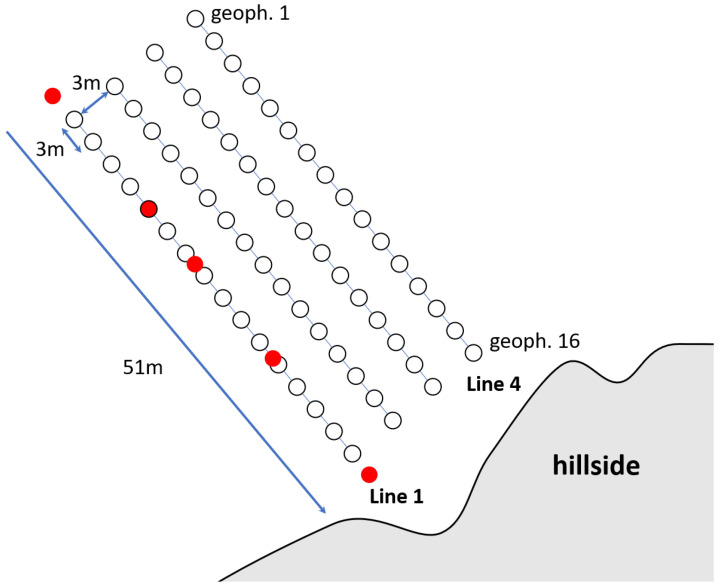
Schematic of the measurement setup in Area A of the gravel pit. White dots indicate the geophone positions, red dots indicate the shot positions, here shown for line array 1 as an example.

For the first measurement, we record seismic data in Area A, which is approximately 10 m below the ground. Figure 4 depicts a closer view of Area A inside the gravel pit. Based on information from the manager of the gravel pit, it is expected that the ground water layer is approximately 2–5 m below the surface. This information serves as ground truth data for our survey. We conduct measurements over a line of 16 geophones with a total length of 51 m. We place the geophones in an equidistant interval of 3 m to each other. To provide better coupling with the gravel in the ground, we drill holes into the ground and dig the geophones into them. In total, we conduct measurements with a single line array that is moved by 3 m perpendicular to its direction. For each measurement, we select five shot positions. At each shot position, we stack the measurements of five shots to increase the signal-to-noise-ratio (SNR) in our data. Table 1 summarizes the survey parameters and the available ground truth information. Figure 5 depicts a schematic view of the measurement setup.

### 2.3. Measurements in Area B of the Gravel Pit

**Figure 6:**
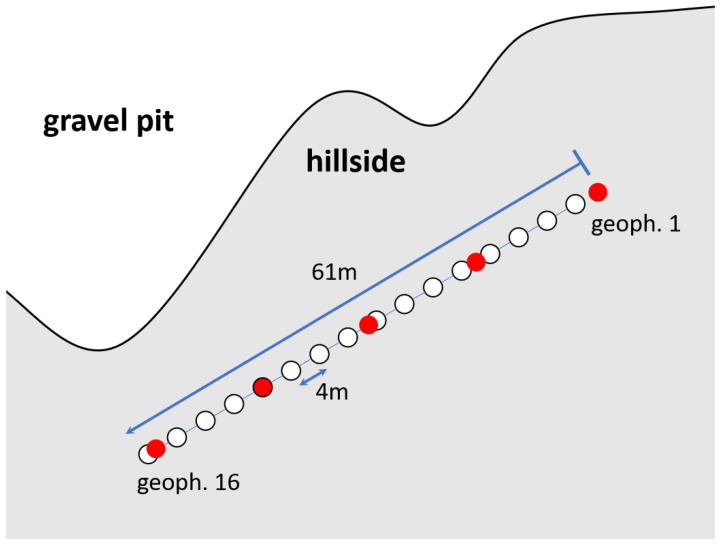
Schematic of the measurement setup in Area B of the gravel pit. White dots indicate the geophone positions, red dots indicate the shot positions.

For the second measurement, we place our geophones above the gravel pit where the subsurface consists more of soil and clay. We therefore expect lower seismic velocities in this area compared to the layers of gravel inside the pit. We conduct measurements for a line array of 16 geophones with a geophone distance of 4 m. Again, we select five shot positions and stack five shots per shot position for a higher SNR. Table 2 summarizes the important survey parameters. Figure 6 depicts a schematic view of the measurement setup.

### 2.4. Inversion Results

To obtain P-wave velocity models of the subsurface for the recorded data, we use the Software SeisImager from Geometrics. The software conducts a travel time tomography based on travel times chosen from the measurement data. The picking of the first-arrival times is performed manually using the software PickWin. After picking the travel times, we generate a two-layer starting model using the time-term inversion [17] contained in the software. After that, based on the starting model, a travel time tomography with 10 iterations is performed. A brief summary of travel time tomography can be found in the Appendix A.

**Figure 7:**
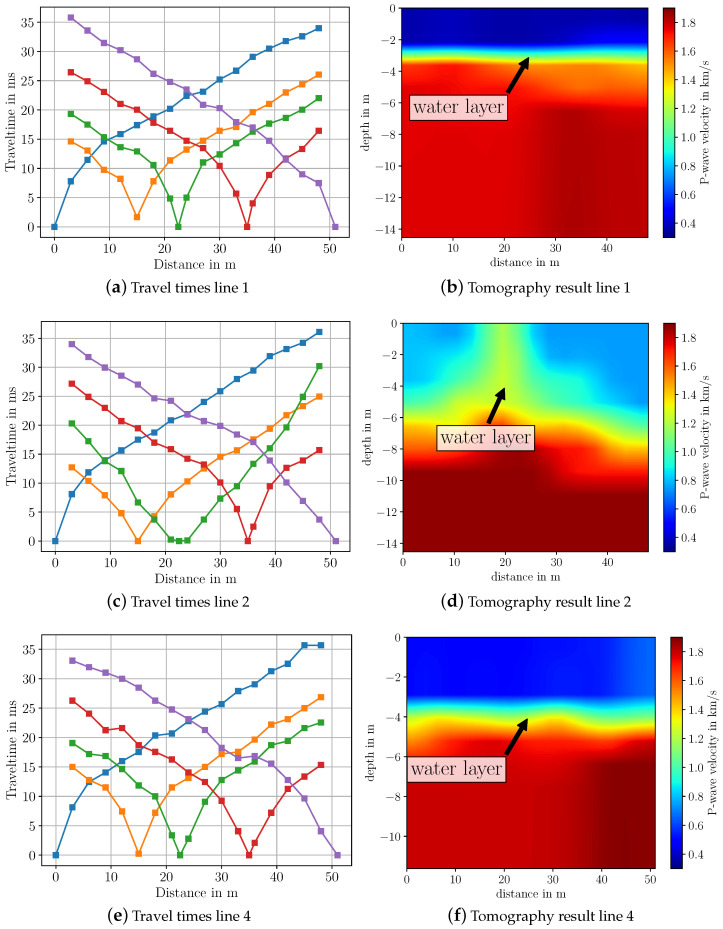
Picked travel time curves and imaging results for measurements in Area A of gravel pit after travel time tomography in SeisImager. Colors in the travel time curves indicate the respective shot.

The results for the measurements inside the gravel pit in Area A are depicted in Figure 7. We notice that measurement data from line 3 are corrupted and therefore cannot be used for an inversion. Hence, results for lines 1, 2 and 4 only are shown. On the left-hand side of Figure 7, the respective travel time curves of the data are shown, while on the right-hand side, the reconstructed P-wave velocity models are depicted. The subsurface models for lines 1 and 4 show a clear two-layer structure with the interface around 3 m to 4 m depth. This can also be seen from the travel time curves that basically consist of two linear functions with different slopes. The slope of the travel time curve is the inverse of the P-wave velocity. Based on ground truth information, the observed interface is very likely to be the transition from a gravel layer to the groundwater layer that was known to be at 2 m to 5 m depth. For line 2, the inversion result looks different: the velocity in the upper level is significantly higher compared to the other results and a clear horizontal layer is not visible. There is a noticeable vertical line of high velocity close to 20 m distance. This behavior can be observed in the travel time curve for the third shot (green line), where travel times around the third shot position at 22.5 m are very low. A reason for this result could be a higher saturation of groundwater in this area that leads to an increased P-wave velocity in the subsurface. During measurements, several locations are observed where the gravel contains more water.

**Figure 8:**
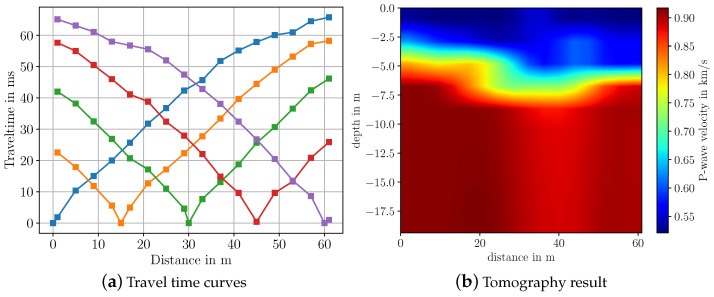
Picked travel time curves and imaging results for measurements in Area B of gravel pit after travel time tomography in SeisImager. Colors in the travel time curves indicate the respective shot.

Figure 8 depicts the travel time curves and the inversion result for the measurements above the gravel pit. Compared to the results from inside the gravel pit (Figure 7), the estimated velocities are much lower. This is due to the fact that there is no groundwater layer close to this location. Hence, the material is less saturated with water. Furthermore, at this location, the material at the surface is mainly soil and partially gravel, which also explains the lower velocities and the smooth velocity gradient over the depth. Again, two distinct layers of differing velocity can be observed corresponding to the travel time curves, where two linear functions with differing slopes are visible for each shot. Compared to the travel times measured in Area A (see Figure 7), the slope of the travel time curves in Area B show a smoother change over the distance. This indicates higher homogeneity in the material, which is expected, since here, no water table is present in the subsurface.

## 3. Seismic Measurements over Highway Tunnel

For the second measurements, we select a surface above the highway tunnel on the highway A99 in Aubing, Germany. The measurements are conducted in December 2021 for half a day.

### 3.1. Measurement Setup

**Figure 9:**
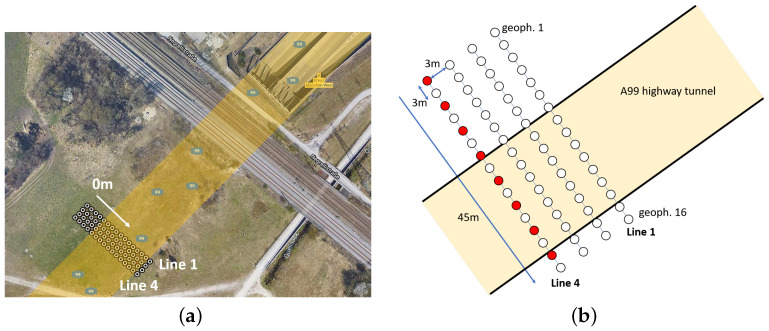
Google Maps and schematic view of the measurement site and setup over the highway tunnel. (**a**) Google Maps data from the measurements over the highway A99 tunnel with geophone positions. The underground highway tunnel is indicated by the yellow shape. (**b**) Schematic of the measurement setup over the highway tunnel. White dots indicate geophone positions, red dots indicate the shot positions, here exemplary for line array 4.

As before, we use one Geode Seismograph with 16 geophones and a sledgehammer as the source. For each measurement stage, we use one line array with all 16 geophones that are placed regularly with a distance of 3 m from each other. The length of the line array is 45 m. We place the line array at four different locations, such that the complete line array has a 3 m distance from its preceding location. For each measurement, we select eight shot positions, where we use four shots at the same position for stacking. Since we have more shot positions compared to the measurements in the gravel pit, we reduce the number of shots for stacking in order to keep the effort for hammer strikes balanced. Figure 9 shows the line array constellation over the highway tunnel with Google Maps data and a schematic of the measurement setup over the highway tunnel. As can be seen, the line arrays are positioned such that a part of the geophones lied over the tunnel, whereas another part continues beyond the extent of the tunnel. This placement is chosen in order to recognize the start of the tunnel in the later inversion results more distinctly. Furthermore, the geophones are placed further away from the railways to record less noise. According to the regional highway management site, the tunnel ceiling within the subsurface is estimated to be approximately 4 m–6 m in depth. Moreover, the start of the tunnel measured from the 0 m position of the geophone line array is approximately 18 m according to Google Maps data. The position of the first and last geophone of each line array is measured in terms of GPS data with the help of a real-time kinematic (RTK) device. The RTK is set up in a base/rover constellation where both the base and rover are equipped with a Piksi Multi GNSS Module from Swift Navigation. For communication between the base and rover, an RFD868 radio modem is used to send and receive telemetry data. We use the rover system to record GPS data at the respective geophone positions. Table 3 summarizes the important survey parameters of each line array for the measurements over the tunnel and the available ground truth information.

### 3.2. Inversion Results

**Figure 10:**
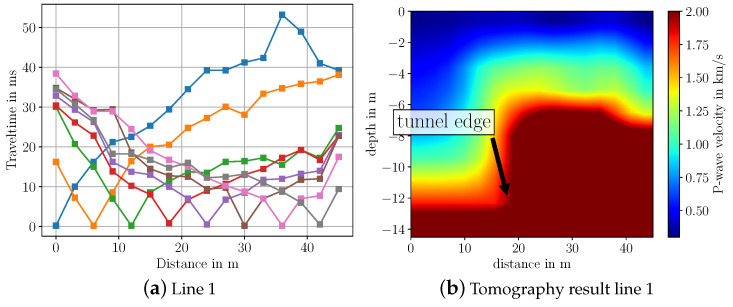
Travel time curves and imaging results from seismic data measured over highway tunnel for line array 1. Colors in the travel time curves indicate the respective shot.

**Figure 11:**
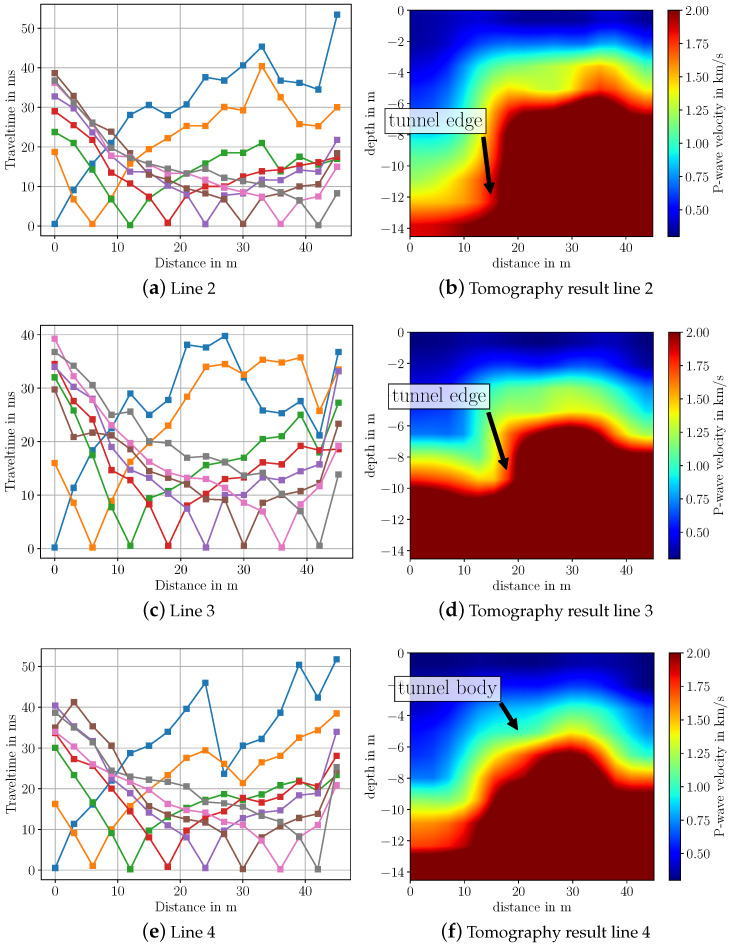
Travel time curves and imaging results from seismic data measured over highway tunnel for line arrays 2, 3 and 4. Colors in the travel time curves indicate the respective shot.

To invert the measurement data, we again pick the first-arrival travel times by hand and then use SeisImager to process the travel time data. The picked travel time curves and the inverted results for the P-wave velocity for all four line arrays are depicted in Figure 10 and Figure 11. From the travel time curves, one can directly see that the subsurface cannot be described by a simple horizontally layered model. Rather, we observe that travel time measurements up to 20 m differ significantly from those between 20 m to 50 m, indicating that we can expect a very different structure in this distance range. It is noticeable that for the first two shot positions at 0 m and 6 m, the travel time curves of all line arrays (blue and orange curves in Figure 10 and Figure 11) differ significantly between 20 m and the end of the line. One reason for this behavior lies in the fact that at these distances, the measured waves have very low amplitude and need to be amplified in order to enable picking of first arrivals. Due to the noise from cars driving inside the tunnel, reliable picking of the first arrival time is challenging to realize.

Regarding the imaging results, especially for line arrays 1 and 2, the tunnel is clearly visible. For line 3 and 4, the tunnel is not as distinctly visible as it is for the other lines. However, it can be still seen that there is a large object in the ground. A possible reason for the worse imaging result is the differing geologic structure at line array 3 and 4 compared to line array 1 and 2. As can be seen from Figure 9a, line array 3 and 4 are closer to the walkway at the bottom border of the picture. At the walkway, a high amount of gravel has been observed. Therefore, we assume that the geologic structure changes from soil to gravel the closer the line arrays are to the walkway. Another reason could be that at line array 3 and 4 more noise from the driving cars in the tunnel is captured. From Figure 10 and Figure 11 one can observe that the travel time curves of line 3 and 4 differ significantly from line 1 and 2. Furthermore, it should be noted that we approximate the measurement surface by a horizontal line, while in reality, the surface has a slope over the tunnel. Hence, the shape of the reconstructed tunnel is not completely horizontal.

Based on Google Maps data and the measured GPS location of the start of each line array, we have ground truth information that the tunnel begins at ≈18 m distance from the starting position of each line array. From the reconstructed subsurfaces in Figure 10 and Figure 11 we observe that especially for line 1, 2 and 3, the starting position of the tunnel matches very well with this ground truth information. Here, the starting position of the tunnel lies between 15 m to 20 m. Regarding the velocities, their values lie in the expected range for saturated and dry sand, which lies between 0.2 km/s–2.0 km/s [18]. For the tunnel, the estimated P-wave velocity lies in the range of 2 km/s to 2.5 km/s. However, for concrete, which is the main material of the tunnel, the usual P-wave velocity lies higher at 3.6 km/s [18]. To summarize, we conclude that we are able to image a large body such as this tunnel using a sledgehammer and geophone equipment.

## 4. Seismic Measurements over Underground Tubes

For the last measurements, we select the premises of the DLR in Oberpfaffenhofen, Germany, with an area where tubes are placed underground. The measurements are conducted in December 2021 for half a day.

### 4.1. Measurement Setup

**Figure 12:**
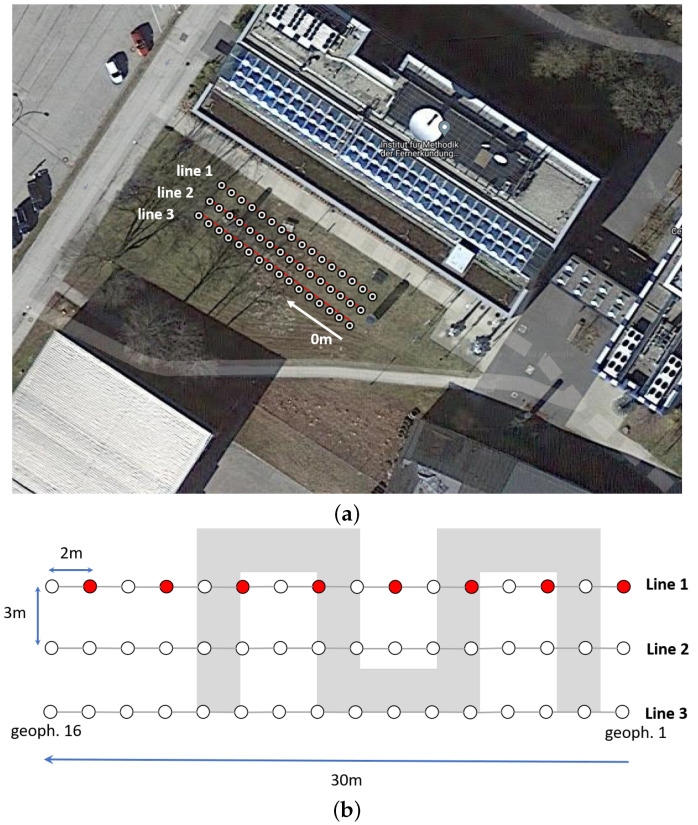
Google maps and schematic view of measurement site over tubes at DLR site. (**a**) Google Maps view with GPS locations of geophones. (**b**) Schematic view of measurement setup with underground tubes indicated in gray. White dots indicate the geophone positions, red dots indicate the shot positions, here exemplary for line array 1.

We use the same equipment as in the preceding measurements, a Geode Seismograph and 16 geophones. We conduct three separate measurements with a line array of 16 geophones each. The line array has a length of 30 m and a distance of 3 m to its preceding location. The geophones are separated by 2 m to each other. For each array, we select eight shot positions, where we use four shots for stacking. According to available ground truth information, the underground tubes are located approximately 1.5 to 2 m below the surface and run in a meandering pattern. This information is based on underground maps that were provided to us by the DLR site management. Table 4 lists the parameters of the measurement setup, Figure 12 shows the corresponding geophone constellation on Google Maps data and a schematic view of the measurement setup over the tubes.

### 4.2. Inversion Results

**Figure 13:**
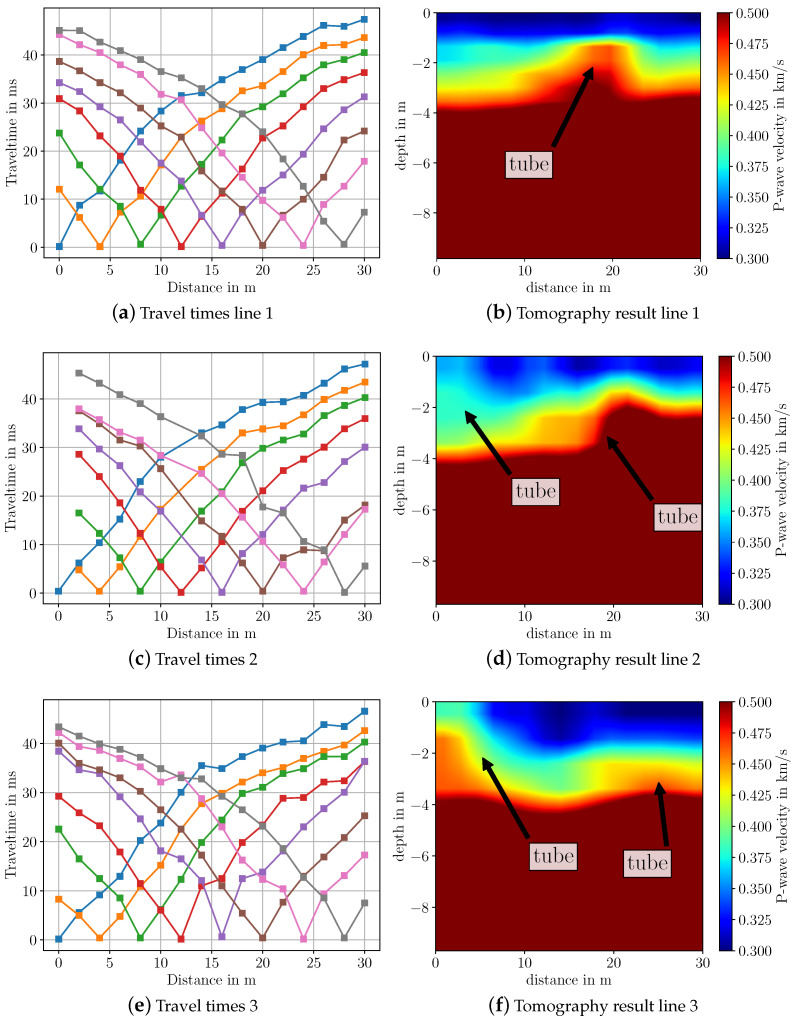
Travel time curves and imaging results from measurements over underground tubes after travel time tomography in SeisImager. Colors in the travel time curves indicate the respective shot.

Figure 13 shows the travel time curves and the corresponding inversion results obtained using travel time tomography in the software SeisImager. In order to see some features in the respective images, the P-wave velocity range has been limited to 0.5 km/s. For all three lines, anomalies of circular shapes can be observed that indicate the locations of the underground tubes. The anomalies start at a depth of 1.5 to 2 m, which indeed coincides with the available ground truth information; see Table 4. According to the available underground map and the schematic view in Figure 12, each line array “cuts” through multiple tubes. Therefore, multiple such features should be visible in the images where all of them lie at a similar distance. This is only partly visible in the images. For line array 1 and 2, the tube feature is at approximately 18 to 20 m distance; see Figure 13b,d. In the image of line array 3, this feature is weakly visible at a distance of 25 m; see Figure 13f. However, here, another feature indicating a tube is visible at a distance of 5 m. The same feature is weakly visible for line array 2 at the same distance; see Figure 13d. As we can observe, it is indeed possible to image certain features of the tubes. Due to the limits of travel time tomography, the spatial resolution is not sufficiently high to visually separate multiple tubes. Applying full waveform inversion could enhance the spatial separation in the images, such that the tubes become more distinctly visible.

## 5. Open Data Set

All measurement data from the campaigns described in the preceding sections are published under https://doi.org/10.6084/m9.figshare.19354334 (accessed on 28 July 2022), and can be used to test and validate seismic data processing and inversion algorithms. The measurement data are stored in the SEG-2 file format developed by the Society of Exploration Geophysicists [19].

**Figure 14:**
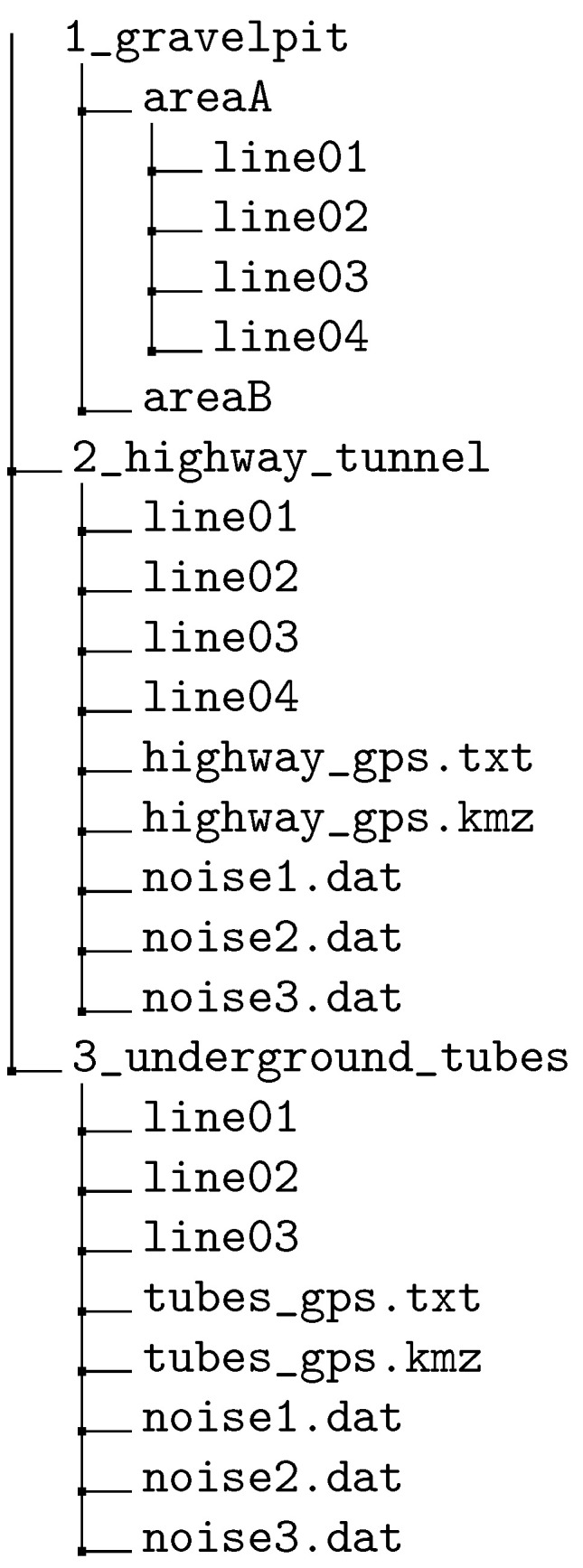
Folder structure of the open seismic data set.

Figure 14 gives an overview of the folder and file structure of the published data set. The data set is structured into three main folders, one for each measurement campaign: 1_gravelpit, 2_highway_tunnel, 3_underground_tubes. In each measurement campaign folder, there are separate subfolders for each line array that contain one SEG-2 file for each shot position. Additionally, for measurements over the highway tunnel and the underground tubes, noise data are recorded at the geophones. To this end, the geophones are placed at the specified positions and seismic data are measured without any active source impact. These files are named noise1.dat, noise2.dat, noise3.dat in the folders 2_highway_tunnel and 3_underground_tubes, respectively. Furthermore, GPS data of the geophone positions are also available for the highway tunnel and underground tubes measurements. These data were obtained by a real-time kinematic (RTK) device that provides accurate GPS coordinates of a specific position. They are saved as a text file with latitude and longitude coordinate of the respective geophone position named highway_gps.txt, tubes_gps.txt, respectively. It should be noted that the GPS coordinates of only the first and last geophone of a line array have been measured using the RTK device. For the remaining positions, the GPS coordinates have been reconstructed using the distance between the geophones. All GPS data are also available as .kmz-files, where the geophone positions can be directly visualized in Google Earth. The files are named highway_gps.kmz, tubes_gps.kmz in the respective campaign folders and can be used, e.g., to recreate Figure 9a and Figure 12a.

## 6. Conclusions

In this paper, we give a detailed description of three near-surface seismic refraction measurements that we conducted in Bavaria, Germany. The motivation to conduct these measurements stemmed from the fact that it is often difficult to obtain seismic data with ground truth information. We therefore recorded measurements at sites where we had ground truth information available in order to verify inversion results with this information. Furthermore, we presented imaging results from our measurements. With the recorded measurement data, we were able to image the ground water level inside the gravel pit at a depth of 2–4 m and a highway tunnel up to a depth of 14 m with sufficient accuracy. In both cases, the resulting images matched well with the available ground truth information about the water level depth and the location of the tunnel. For the underground tubes, we were able to image certain features of the tubes at depths of 2 m. This also matched available ground truth information about the depth location of the tubes. However, the spatial resolution of the obtained images is limited, such that detailed visual separation of multiple tubes over the horizontal axis was not possible. More advanced imaging techniques such as the full waveform inversion will probably enhance the spatial resolution around the tubes.

As mentioned in the introduction, GPR serves as an alternative or additional technique to seismic refraction for near-surface imaging. For imaging the underground tubes, GPR will probably achieve better results with an improved resolution of the tubes, since these are very close to the surface and provide clear permittivity jumps from the surrounding soil due to their material. In case of the water layer in the gravel pit, GPR should also produce adequate images of the material layers. Since the gravel was very dry during the measurements in summer, GPR should reach depths of several meters and should therefore be able to image the water layer. For the highway tunnel, GPR’s penetration depth could be rather limited due to moisture and clay within the soil. Thus, GPR would not be able to capture the tunnel, particularly the deeper tunnel edge.

All seismic sensors that we used for the campaigns were cable based. Hence, deploying the geophones for each survey consumed a lot of time. Here, nodal seismic systems with wireless solutions where geophones are connected to a central data grabber via wireless connections will definitely ease the execution of the measurements. Such solutions already exist on the market (see, e.g., Wireless Seismic Inc., NuSeis, SmartSolo etc.) and would be a better choice for near-surface seismic surveys with hammer strikes that need to be conducted in an easy and fast manner. Such wireless solutions also provide direct GPS data of their seismic nodes, another feature that eases conduction of measurements and later data processing.

From our field measurements and imaging results, we conclude that seismic refraction surveys with basic equipment such as a hammer, geophones and a data collector such as the Geometrics Geode can indeed be used to image near-surface structures. However, based on our results, it is more suited for imaging structures that do not lie in the direct vicinity of the surface. This became apparent in the case of the underground tubes which lay 1–2 m below the surface. In such cases, the probability is high that direct and refracted waves interfere with each other, such that no separation of these signals is possible. Thus, resolving structures in this shallow regime will be challenging using seismic techniques and GPR would be a better choice. Furthermore, even for a large, distinct body such as the highway tunnel, seismic imaging based on hammer strikes might not give sufficiently accurate images of the structures. To achieve higher accuracy, a more powerful energy source such as a falling weight or vibrating sources is necessary. Nevertheless, to obtain a rough estimate of near-surface structures, a seismic refraction survey with hammer strikes is a suitable choice.

## Figures and Tables

**Table 1:** Survey setup for measurements in Area A of the gravel pit.

Survey Parameter	Parameter Value
Array length	51 m
No. of shots	5
Shot positions	{0, 15, 22.5, 35, 51} m
Stacks/shot	5
Geophone distance	3 m
Geophone positions	{3, 6, 9, 12, 15, 18, 21, 24, 27, 30, 33, 36, 39, 42, 45, 48} m
Sampling interval	0.25 m s
Trace length	200 ms
**Ground truth info:**	Ground water layer at 2 m–5 m depth

**Table 2:** Survey setup for measurements in Area B of the gravel pit.

Survey Parameter	Parameter Value
Array length	61 m
No. of shots	5
Shot positions	{0, 15, 30, 45, 60} m
Stacks/shot	5
Geophone distance	4 m
Geophone positions	{1, 5, 9, 13, 17, 21, 25, 29, 33, 37, 41, 45, 49, 53, 57, 61} m
Sampling interval	0.25 m s
Trace length	200 ms

**Table 3:** Survey setup for measurements over highway tunnel.

Survey Parameter	Parameter Value
Array length	45 m
No. of shots	8
Shot positions	{0, 6, 12, 18, 24, 30, 36, 42} m
Stacks/shot	4
Geophone distance	3 m
Geophone positions	{0, 3, 6, 9, 12, 15, 18, 21, 24, 27, 30, 33, 36, 39, 42, 45} m
Sampling interval	0.25 m s
Trace length	400 ms
**Ground truth info:**	Tunnel ceiling at 4 m–6 m depth
	Tunnel start at 18 m distance

**Table 4:** Survey setup for measurements over underground tubes.

Survey Parameter	Parameter Value
Array length	30 m
No. of shots	8
Shot positions	{0, 4, 8, 12, 16, 20, 24, 28} m
Stacks/shot	4
Geophone distance	2 m
Geophone positions	{0, 2, 4, 6, 8, 10, 12, 14, 16, 18, 20, 22, 24, 26, 28, 30} m
Sampling interval	0.125 m s
Trace length	300 ms
**Ground truth info:**	Underground tubes at 1.5 m–2 m depth

## Data Availability

All measurement data from the campaigns described in the preceding sections are published under https://doi.org/10.6084/m9.figshare.19354334 and can be used to test and validate seismic data processing and inversion algorithms. The data set contains one file in SEG-2 format for each shot position where all measurements conducted over one line array are contained in one folder.

## Abbreviations

The following abbreviations are used in this manuscript:

DLR	German Aerospace Center
GPS	Global positioning system
GPR	Ground penetrating radar
RTK	Real-time kinematic

## Appendix A. Brief Summary of Travel Time Tomography

In the following, we give a short summary of the travel time tomography for seismic imaging since in the upcoming sections it is used to obtain subsurface images from our measurement data. Travel time tomography is a geophysical imaging technique that relies on the first-arrival times of seismic waves. Hence, it considers the time a wave travels from source to receiver. In mathematical terms, it is based on the minimization of a squared residual between measured first-arrival travel times $T_{\mathrm{obs},r}$ and synthesized travel times $T_{\mathrm{syn},r}\left(m\right)$ based on a subsurface model m at receiver *r* for a total of *R* receivers:

(A1) $$\underset{m}{\min\;}J\left(m\right)=\frac{1}{2}\sum_{r=1}^{R}{\left|T_{\mathrm{syn},r}\left(m\right)-T_{\mathrm{obs},r}\right|}^{2}.$$

The variable m is a subsurface parameter of interest and an explicit function of the spatial coordinate x. In our case, it is the spatial distribution of the P-wave velocity in the subsurface. To evaluate the cost in (A1), synthetic travel times need to be computed. To this end, the eikonal equation with respect to the spatial coordinate x can be used:

(A2) $$\left|\nabla T\left(x\right)\right|=\frac{1}{m(x)}.$$

By solving (A2) for T(x) one obtains a spatial travel time map over x with the first-arrival times of a wave that originates from a source point. The eikonal equation is a partial differential equation and different numerical methods exist for solving it. For instance, the fast marching method [20], fast sweeping method [21] or the fast iterative method [22] can be used for this purpose. By solving (A2) we can generate synthetic travel time data $T_{\mathrm{syn},r}\left(m\right)$ in the cost (A1) for all receivers. Expression (A1) is minimized with respect to the subsurface parameter m. To this end, a gradient descent method is used which requires computation of $\frac{\partial }{\partial m}J\left(m\right)$. This can be achieved by using either ray tracing methods [23] or the adjoint state method [24,25]. After obtaining $\frac{\partial }{\partial m}J\left(m\right)$ a gradient descent step can be used to update the subsurface model m and a new gradient can be computed based on this updated model. These two steps are conducted in an iterative manner until the cost J(m) is minimized and an adequate subsurface model has been estimated.

The eikonal Equation (A2) can be derived from the scalar wave equation by assuming only high-frequency solutions [26]. Doing so simplifies the wave physics by ray theory and therefore reduces computational burden in the forward solution. However, this high-frequency approximation also has consequences for the achievable spatial resolution of the tomography method. More specifically, by using ray theory instead of wave theory the spatial resolution of the travel time tomography is limited by the first Fresnel zone [27]. Spatial objects below this limit cannot be resolved by travel time tomography and hence, the obtained images are usually of very smooth nature lacking high-frequency spatial details. On the other hand, travel time tomography is computationally less complex compared to the full waveform inversion that exploits the wave equation. It is therefore usually employed to generate a rough image of the subsurface that is then refined by full waveform inversion [28].
